# Increased hepatitis C virus co-infection and injection drug use in HIV-infected fishermen in Myanmar

**DOI:** 10.1186/s12879-018-3558-y

**Published:** 2018-12-14

**Authors:** Janet Ousley, Robin Nesbitt, Nang Thu Thu Kyaw, Elkin Bermudez, Kyi Pyar Soe, Rey Anicete, Phyu Ei Mon, Win Le Shwe Sin Ei, Susannah Christofani, Marcelo Fernandez, Iza Ciglenecki

**Affiliations:** 1 MSF-Switzerland, Myanmar Project, #101 Dhama Zedi Road, Kamayut Tsp, Yangon, Myanmar; 2 MSF, Geneva, Switzerland; 3 Medecins Sans Frontiers, 333 7th Avenue, 2nd Floor, New York, NY 10001 USA

**Keywords:** Migration, Risk behaviors, Southeast Asia, Epidemiology-HIV/AIDS

## Abstract

**Background:**

In Southeast Asia, though fishermen are known to be a key population at high risk of HIV, little is known about their co-infection rates with Hepatitis C virus (HCV), or how illness and risk behaviors vary by occupation or type of fishermen. In Myanmar, this lack of knowledge is particularly acute, despite the fact that much of the country’s border is coastline.

**Methods:**

We conducted a retrospective analysis to assess clinical, demographic, and risk characteristics of HIV-infected, ≥15-year-old males under HIV care from 2004 to 2014. Subgroups of fishermen were categorized according to the location of fishing activities, boat ownership, and length of time at sea. Generalized linear models assessed odds of high risk behaviors, including MSM (men who have sex with men), transactional sex, injection drug use (IDU), and HCV co-infection among international, local subsistence, and national migrant fishermen.

**Results:**

Of 2798 adult males who enrolled in HIV care between 2004 and 2014, 41.9% (*n* = 1172) were fishermen. Among these, migrants had the highest odds of engaging in risk behaviors such as sex work (Myanmar national migrants: OR 3.26 95% CI: 2.20 to 4.83), and injecting drugs (international migrants: OR 2.93, 95% CI: 1.22 to 3.87) when compared to the general male HIV clinic population. 15.9% of all fishermen reported past or current IDU (23.0% of international migrants). 22.8% of all fishermen were also co-infected with HCV, and though predictably injectors had the highest odds (OR 20.1, 95% CI: 13.7 to 29.5), even after controlling for other risk factors, fishermen retained higher odds (OR 2.37 95% CI: 1.70 to 3.32).

**Conclusions:**

HIV positive fishermen in Myanmar had higher odds of HCV co-infection. They also disproportionally injected drugs and engaged in transactional sex more than other patients. This is especially pronounced among international migrant fishermen. HIV-infected fishermen should be counseled on high risk activities, screened for HCV, and targeted by harm reduction programs.

## Background

Due to the mobile nature of seafaring, fishing industry communities’ close sexual networks, and their relative access to cash and income (and often, thus, the frequency with which they engage in transactional sex), fishermen have long been recognized as vulnerable to HIV infection [1–4]. In Southeast Asia, where fisheries employ an estimated 30 million people and supply a quarter of global fish production annually, a prior review found HIV seroprevalence rates among fishing communities to be between 4.6 and 14 times higher than in the general population, ranging from 15.5% in Thailand (*n* = 818), 16.2% in Cambodia (*n* = 465), and 12.4% in Malaysia (*n* = 398) [5–8]. In Myanmar, despite an abundance of coastline and fishing related economic activity, fishermen remain mostly unmonitored by HIV surveillance systems, international organizations, and national AIDS programming [9]. What’s more, the very behaviors that leave fishermen susceptible to HIV infection may also expose them to other infections such as hepatitis C virus (HCV), particularly among injection drug users [10]. This study describes the clinical, demographic, and treatment characteristics of HIV-infected fishermen attending an MSF HIV clinic, and compares their risk characteristics with non-fishermen HIV-infected males at the same clinical site. It is the first to look at this patient population in Myanmar.

## Methods

### Study design

A retrospective cohort analysis was conducted using a convenience sample of anonymized medical data for all patients presenting between 2004 and 2014.

### Study context

Medecins Sans Frontiers (MSF) has been operating an HIV clinic in Dawei District, Tanintharyi Division, in southern Myanmar, since 2004. One of the two largest fish producing areas in the country, Tanintharyi generated 650,000 tons of fish during the 2009–2010 seasons, over twice the yield of the next most productive state, according to recent data [11]. The entire Western border of the region is coastline bordering the Andaman sea, and much of the population lives along this coast or along multiple rivers and tributaries, providing ample opportunities for fishing both within Myanmar waters and in the international waters off of Thailand and beyond [12]. MSF is providing HIV testing, treatment and care for high risk groups, including those working in fishing related industries.

All data used in this analysis were gathered as part of routine clinical assessments at the MSF facility and research procedures were in accordance with the Declaration of Helsinki. Analysis fulfilled the exemption criteria set by the Médecins Sans Frontières Ethics Review Board for a posteriori analyses of routinely collected, de-identified clinical data and thus did not require ERB review, nor did it require Myanmar National ERB approval as a review of anonymized programmatic information [13].

### Study population

All adult male (≥15 years) HIV positive patients who accessed HIV care at the MSF Dawei clinic from 2004 to 2014 were included. For the purposes of our study, a fisherman was defined as a person who, upon presentation to care, self-reported catching fish as their primary income generating activity (though were not necessarily involved in the resale, transfer, or processing of fish). Occupation was established during an interview at the initial clinic visit, and data were de-identified using an anonymous patient number. Three subgroups of fishermen existed and were categorized according to the location of fishing activities, boat ownership, and length of time at sea: 1) local subsistence fishermen worked on smaller boats, owned by themselves or another Myanmar citizen, and spent shorter lengths of time at sea, sometimes even returning home the same day; 2) Myanmar migrant fishermen were those whose fishing occurred further away from Dawei along Myanmar’s coastline and may have spent somewhat longer lengths of time at sea, a few days to a few weeks, also in a vessel owned by a Myanmar citizen; and 3) international migrant fishermen worked on boats owned by a foreigner, often deep-sea vessels traveling in international waters, and often spending months away from home.

### Statistical analyses

Baseline demographic and treatment characteristics were compared between fishermen and non-fishermen patients using means with their standard deviations (SD), or medians with corresponding interquartile ranges (IQR) for continuous variables, and frequencies and proportions for categorical data. Tests for differences between fishermen and the general male cohort were performed using Mann-Whitney U tests or t-tests for continuous variables and chi squared tests for categorical variables.

Three behavioural risk factors for HIV were investigated in the total male cohort enrolled in HIV care (i.e. those who initiated antiretroviral therapy as well as those who do not): transactional sex with a commercial sex worker (CSW); men who have sex with men (MSM); and intravenous drug use (IDU). Odds ratios for these factors were analyzed using generalized linear models with binomial distribution and robust standard errors. Risk factors for HCV co-infection (expressed as positive HCV seroprevalence using Oraquick Rapid Antibody Test) were investigated amongst patients who had been tested whilst on ART, using the same statistical procedures; these included clinical and demographic characteristics (age, BMI, CD4 count, marital status, WHO stage) as well as behavioural risk factors for HIV.

## Results

### Demographic characteristics

**Fig. 1:**
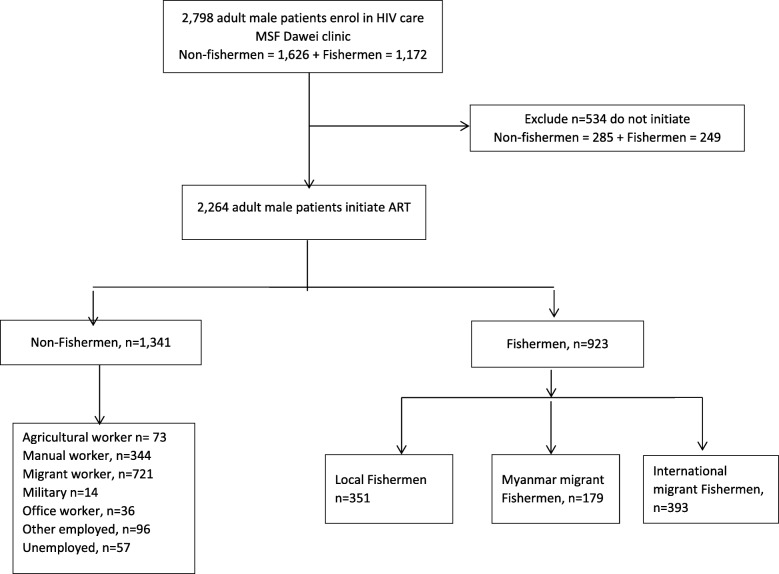
Patients included in analysis

There were a total of 2798 HIV positive adult males who accessed HIV care at the MSF clinic from July 2004 until December 2014 (Fig. 1). Amongst those enrolled in HIV care, fishing was reported as the most common occupation (*n* = 1172, 41.9%). Other migrant work (non-fishing; 31.0%) and manual workers (14.8%) were also common. Of the fishermen who enrolled in HIV care, 453 (38.6%) were local subsistence fishermen, 239 (20.4%) were Myanmar migrants and 480 (41.0%) were international migrant fishermen. There was no difference in mean age at enrolment in HIV care between fishermen and non-fishermen (36.9 and 37.1 years respectively, *p* = 0.6), although a higher proportion of fishermen were 30–49 years (Table 1). Fishermen and non-fishermen differed in their marital status, with fewer fisherman married (51.6% vs 58.6%, *p* = 0.003).

**Table 1:** Baseline characteristics of male HIV+ adults at enrolment in HIV care at Dawei clinic- n=2798^1^

Characteristics		General (*n* = 1626)		Fishermen (*n* = 1172)		*p*-value
		*n*	%	*n*	%	
Age (years)	15–29	355	21.7	191	16.3	< 0.001
	30–49	1141	70.3	906	77.3	
	50+	130	8.0	75	6.4	
	Mean (sd)	37.1	(8.8)	36.9	(7.5)	0.6
Marital status	Married	952	58.6	605	51.6	0.003
	Single	427	26.3	350	29.9	
	Divorced	140	8.6	127	10.8	
	Widow	107	6.6	90	7.68	
BMI	> 18.5^2^	791	48.7	598	51.0	0.001
	< 18.5	699	43.0	435	37.1	
	Missing	136	8.4	139	11.9	
	Median (IQR)	18.7	(17–20.5)	19.0	(17.4–20.6)	0.01
CD4 count	< 50	415	25.5	292	24.9	0.025
	50	421	25.9	305	26.0	
	200	173	10.6	105	9.0	
	350	89	5.5	52	4.4	
	500	83	5.1	41	3.5	
	Missing	445	27.4	377	32.2	
	Median (IQR)	90	(30–233)	84	(39–198)	0.18
WHO Stage	1	165	10.2	73	6.2	0.004
	2	235	14.5	176	15.0	
	3	833	51.2	633	54.0	
	4	393	24.2	290	24.7	
Risk factors for HIV acquisition	IDU	138	8.49	186	15.9	< 0.001
	CSW	1086	66.9	980	83.7	< 0.001
	MSM	157	9.7	47	4.02	< 0.001
Initiate ART		1341	82.5	923	78.8	0.014
HCV positive ^3^		93	9.5	154	23.2	< 0.001

^1^Total includes *n* = 2 non-fishermen who are < 15 at enrolment but > 15 at ART initiation, *p*-values from chi square or Mann-Whitney U test for BMI and CD4 count and t-test for age; ^2^ this category includes 37 patients with BMI > 24.9 and 3 patients with BMI > 30; ^3^ amongst 1648 screened for HCV. *ART = antiretroviral therapy

### High risk behaviour

Male fishermen in care engaged in high-risk activities more than non-fishermen enrolled in HIV care. Fishermen had 2.5 times higher odds of buying sex from a CSW than the general cohort (OR 2.53 95% CI: 2.09 to 3.06), the highest odds among Myanmar migrant fishermen (OR 3.26 95% CI: 2.20 to 4.83), of whom 87% reported buying sex from a CSW (Table 2). In addition, fishermen had almost 90% higher odds of current or past IV drug use than non-fishermen (OR 1.89 95% CI: 1.49 to 2.39). This was driven by the higher prevalence of drug use in international fishermen, who had almost three times the odds of IDU in adjusted models (OR 2.93, 95% CI: 2.22 to 3.87) and among whom 23% reported past or current drug use, compared to 13% of local fishermen and 8% of both non-fishermen and Myanmar migrant groups. There were lower odds of MSM among fishermen compared to non-fishermen (OR 0.34, 95% CI: 0.23 to 0.48), also in all fishermen subgroups.

**Table 2:** Crude and adjusted odds ratios for MSM, CSW, IDU in male HIV+ adults at enrolment in HIV care at Dawei clinic (include those who initiate and do not initiate ART)- comparing fishermen and fishermen subtypes to the general non-fishermen cohort

	MSM (*n* = 2781)					CSW (*n* = 2793)					IDU (*n* = 2974)				
		Crude		Adjusted			Crude		Adjusted			Crude		Adjusted	
	%	OR	95% CI	OR	95% CI	%	OR	95% CI	OR	95% CI	%	OR	95% CI	OR	95% CI
Non-fishermen	9.7	1		1		66.9	1		1		8.5	1		1	
Fisherman	4.0	0.39	0.28–0.54	0.34	0.23–0.48	83.7	2.53	2.10–3.05	2.53	2.09–3.06	15.9	2.03	1.61–2.57	1.89	1.49–2.39
Non-fishermen	9.7	1		1		66.9	1		1		8.5	1		1	
Local	3.5	0.34	0.20–0.57	0.29	0.17–0.50	79.3	1.88	1.47–2.42	1.91	1.48–2.46	12.6	1.55	1.12–2.15	1.49	1.07–2.06
International	3.8	0.36	0.22–0.60	0.31	0.18–0.52	86.2	3.09	2.33–4.09	3.07	2.31–4.08	23.0	3.21	2.44–4.22	2.93	2.22–3.87
Myanmar	5.5	0.54	0.30–0.96	0.49	0.26–0.91	87.0	3.31	2.24–4.90	3.26	2.20–4.83	8.0	0.93	0.56–1.53	0.85	0.52–1.41

% with outcome (MSM, CSW, IDU) Reference group is always general non-fishermen cohort; adjusted models include age at enrolment and marital status

### Clinical, immunological, and treatment characteristics

At presentation, fishermen had a slightly higher median BMI (19.0 IQR 17.4–20.6) than non-fishermen (18.7 IQR 17–20.5, *p* = 0.01), though no statistical difference in CD4 count between the two groups (90 IQR 30–233 vs 84 IQR 39–198, *p* = 0.18 Table 1). Slightly more fishermen enrolled later in HIV care than non-fishermen (78.8% WHO stage 3/4, vs 75.4%, *p* = 0.038), failed to initiate ART (78.8 vs 82.5%, *p* = 0.014), and had a longer median time to ART initiation (42 days vs 32 days, p = < 0.01).

### Hepatitis C virus (HCV) CO-INFECTION

Of the HIV patients on ART, 1648 (72.8%) were screened for HCV, with equal proportions screened from both occupational groups (72.1% fishermen vs 73.3% non-fishermen, *p* = 0.51). Significantly more fishermen were HCV seropositive than non-fishermen (23.2% vs 9.5%, *p* < 0.001), with 28.5% of international fishermen and 21.5% of local fishermen screening positive for the disease, respectively, corresponding to 2.4 times higher odds of HCV co-infection compared to the general cohort in adjusted models (Table 3). When disaggregated by fisherman subtype, local and international fishermen had 2.6 times higher odds of HCV co-infection. The biggest risk factor for HCV infection was past or current IV drug use, and 67% of adult males on ART who reported IDU also screening HCV seropositive. IDU was associated with a 20-fold increased odds of infection (OR 20.1, 95% CI: 13.7 to 29.5), though even after accounting for IDU in an adjusted model, fishermen retained higher odds of HCV infection (OR 2.37, 95% CI: 1.70–3.32, Table 3, adjusted model 1).

**Table 3:** HCV co-infection in HIV + males initiated on ART at MSF clinic in Dawei- 2004-2014, *n* = 1648

Characteristics		HCV+	Crude *n* = 1648		Adjusted model 1 *n* = 1639		Adjusted model 2 *n* = 1639	
		%	OR	95% CI	OR	95% CI	OR	95% CI
Occupation	Non-fisherman	9.5			1			
	Fisherman	23.2	2.88	2.18–3.81	2.37	1.70–3.32		
Fisherman sub-groups	Non-fisherman	9.5						
	Local	21.5	2.62	1.80–3.81			2.66	1.75–4.04
	International	28.5	3.82	2.73–5.34			2.59	1.70–3.96
	Myanmar	15.1	1.7	1.02–2.84			1.50	0.77–2.91
Age at initiation	15–29 yrs	7.2			1			
	30–49 yrs	17.2	2.68	1.65–4.38	2.26	1.40–3.65	2.25	1.39–3.63
	50+	8.4	1.18	0.53–2.63	1.59	0.70–3.64	1.59	0.69–3.65
Marital status	Married	13.2						
	Single	19.1	1.56	1.15–2.10	1.23	0.84–1.79	1.23	0.84–1.79
	Divorced	16.1	1.26	0.78–2.05	0.9	0.50–1.64	0.92	0.50–1.67
	Widow	11.8	0.88	0.48–1.62	0.9	0.41–1.93	0.9	0.42–1.93
BMI at initiation	< 18.5	12.4						
	> 18.5	16.2	1.37	0.90–2.08	1.21	0.71–2.06	1.22	0.72–2.09
	Missing	15.1	1.26	0.84–1.88	1.25	0.73–2.14	1.23	0.72–2.12
CD4 count at initiation	< 50	14.7						
	50	17.9	1.27	0.85–1.89	1.49	0.88–2.53	1.55	0.91–2.64
	200	7.7	1.03	0.71–1.49	0.86	0.41–1.83	1.26	0.74–2.16
	350	16.9	0.49	0.26–0.91	2.01	0.92–4.37	0.86	0.40–1.83
	500	18.9	1.18	0.61–2.31	1.29	0.40–4.20	2.02	0.92–4.44
	Missing	15.0	1.36	0.56–3.26	1.29	0.76–2.18	1.35	0.42–4.42
WHO Stage	1/2	8.9	1		1			
	3/4	16.1	1.98	1.26–3.11	1.45	0.84–2.50	1.46	0.85–2.52
Risk factors for HIV acquisition^a^	IDU	67.0	22.81	15.99–32.54	20.1	13.69–29.52	19.73	13.45–28.94
	MSM	5.7	0.32	0.16–0.66	0.45	0.20–1.01	0.46	0.21–1.03
	CSW	16.2	1.45	1.05–2.02	1.00	0.67–1.50	1.02	0.68–1.53

Population includes only n = 1648 screened for HCV during follow up and excludes 534 who did not initiate ART. Model 1 compares fishermen to general non-fishermen cohort. Model 2 compares fishermen subgroups to general cohort. ^a^For each risk factor, baseline for odds ratio is those without risk factor

## Discussion

This analysis establishes the clinical, demographic, and risk characteristics of a cohort of adult HIV patients about which, to date, no information has been published in Myanmar or in Southeast Asia. Occupations that demand mobility or migration are, by definition, less stable. In this case, they may also hold more health risks: Fishermen often have little access to health facilities and sources of health information while at sea, and they may be socially marginalized and endure real physical risks as part of their work. Weekly, seasonal, or long term migration is an expected part of the occupation, and close knit peer networks replacing family and community while far from home can increase the frequency of unsafe activities fishermen engage in [8].

Our results confirm that Myanmar fishermen also lead lifestyles suffused with risk, yet remain largely untargeted as a subgroup by social services and health and risk education providers in the country. Over 80% of fishermen under MSF’s care reported engaging in commercial sex (especially common in migrants away from their homes and families), which was particularly troubling since past meta-analyses have found that up to 90% of fishermen use condoms infrequently with their primary partners [14]. High risk IDU, especially among international fishermen, is also troubling in the world’s second largest opium producer (Myanmar is home to the notorious “Golden Triangle,” a major drug producing region that spans Myanmar, Laos, and Thailand) [15]. For certain occupations in Myanmar, such as miners in the north of the country, drug use is understood to be a problematic driver of morbidity, and programs target these groups with harm reduction and HIV interventions accordingly [9]. Yet fishermen in the south of the country fall almost entirely outside of these efforts, and national HIV surveillance data largely overlooks them and the Tanintharyi region in strategic documents [9]. Because of the retrospective, clinical nature of the data available for this analysis, an in-depth investigation of these factors was unavailable for this cohort and are worthy of future research. However our results make clear that HIV and harm reduction actors in Myanmar would do better to coordinate their activities to target fishermen with these programs.

Furthermore, risk factors such as IDU may be leading to increased proportions of HCV seroprevalence among fishermen. Rates seen in this analysis were higher than those found in other parts of Myanmar [16]. This is a serious issue. HCV therapies are not yet widely available in Myanmar, especially to vulnerable or indigent populations, and official guidance has only recently recommended using Direct Acting Anti-retrovirals (DAAs) rather than older, less expensive interferon based therapies that can interact with ART. Recent evidence has also suggested that people co-infected with HIV and HCV have a higher incidence of other illnesses, such as an almost two-fold risk of developing cancer [17]. The HIV/HCV co-infection rates seen in these fishermen, as well as the inter-related patterns of fishing, injecting drugs, and transactional sex, warrant further in depth study to better target HIV and HCV prevention and screening efforts, as well as better treatment of both diseases.

### Limitations

Some risk behavior related information may have been underestimated as 1) self-reported information about high risk sex and drug use is sensitive in the Myanmar cultural context, 2) in some cases, patients engaging in multiple high risk behaviors may not have had all their risk factors captured in clinic databases 3) risk information was asked about during initial clinic visits and may not account for changes in behavior during follow up. As with all operational research, some subtle inconsistencies in reporting due to staff transition may have occurred over a decade of data collection, particularly distinctions between types of fishermen.

## Conclusion

In Southeast Asia, the HIV epidemic is not generalized, but resides within specific higher risk groups. This analysis shows that in Myanmar, outside of the traditional CSW, MSM, and IDU risk group categories, fishermen disproportionally inject drugs, are HCV co-infected, and engage in transactional sex. Since many of these men are migrating internationally, they are of note to HIV actors across the region. A better understanding how lengths of time at sea and destinations may affect their risk for disease transmission could better target this group. Introducing systematic HCV screening and harm reduction services for fishermen in Myanmar could be important to curbing both the HIV and HCV epidemics there.

## Abbreviations

AIDS: Acquired Immunodeficiency Syndrome
ART: Anti-Retroviral Therapy
CD4: A glycoprotein in t-cells which are counted as an indicator of immune health
CI: Confidence Interval
HCV: Hepatitus C Virus
HIV: Human Immunideficiency Virus
IQR: Inter-Quartile Range
MSF: Medecins Sans Frontieres
OR: Odds Ratio
TB: Tuberculosis
